# Correlation between structure and function in phosphatidylinositol lipid–dependent Kir2.2 gating

**DOI:** 10.1073/pnas.2114046119

**Published:** 2022-03-14

**Authors:** Yuxi Zhang, Xiao Tao, Roderick MacKinnon

**Affiliations:** ^a^ Laboratory of Molecular Neurobiology and Biophysics, HHMI, The Rockefeller University, New York, NY ,10065

**Keywords:** inward rectifier, phosphatidylinositol lipid, single-channel analysis, PI(4,5)P2, PI(4)P

## Abstract

Inward rectifier K^+^(Kir) channels regulate cell membrane potential. Different Kir channels respond to unique ligands, but all are regulated by phosphatidylinositol 4,5-bisphosphate (PI(4,5)P_2_). Using planar lipid bilayers, we show that Kir2.2 exhibits bursts of openings separated by long quiescent interburst periods. Increasing PI(4,5)P_2_ concentration shortens the Kir2.2 interburst duration and lengthens the burst duration without affecting dwell times within a burst. From this, we propose that burst and interburst durations correspond to the cytoplasmic domain (CTD)–docked and CTD-undocked conformations observed in the presence and absence of PI(4,5)P_2_ in atomic structures. We also studied the effect of different phosphatidylinositol lipids on Kir2.2 activation and conclude that the 5′ phosphate is essential to Kir2.2 pore opening. Other phosphatidylinositol lipids can compete with PI(4,5)P_2_ but cannot activate Kir2.2 without the 5′ phosphate. PI(4)P, which is directly interconvertible to and from PI(4,5)P_2_, might thus be a regulator of Kir channels in the plasma membrane.

## Significance

Phosphatidylinositol 4,5-bisphosphate (PI(4,5)P_2_) levels regulate cell membrane voltage by gluing two halves of a K^+^ channel together and opening the pore. PI(4)P competes with this process. Because both of these lipids are relatively abundant in the plasma membrane and are directly interconvertible through the action of specific enzymes, they may function together to regulate channel activity.

Inward rectifier K^+^ (Kir) channels are so named because they conduct K^+^ better at negative membrane potentials, allowing them to affect “resting” potential while minimizing K^+^ outflow during depolarization (1–6). Kir channels are involved in many physiological processes, including the regulation of cell membrane potential, cellular pacemaker activity, and hormone secretion (3, 7). Structurally, Kir channels comprise four subunits, each containing a transmembrane domain (TMD) with a selectivity filter and a cytoplasmic domain (CTD) connected to the TMD by a linker (8, 9). While different subclasses of Kir channels are regulated by unique modulators, for example, G proteins in the GIRK channel and adenosine triphosphate (ATP) in the ATP-sensitive potassium (KATP) channel, all Kir channels are regulated by phosphatidylinositol 4,5-bisphosphate (PI(4,5)P_2_), a signaling lipid present in the cell plasma membrane (7, 10–15).

**Fig. 1.**
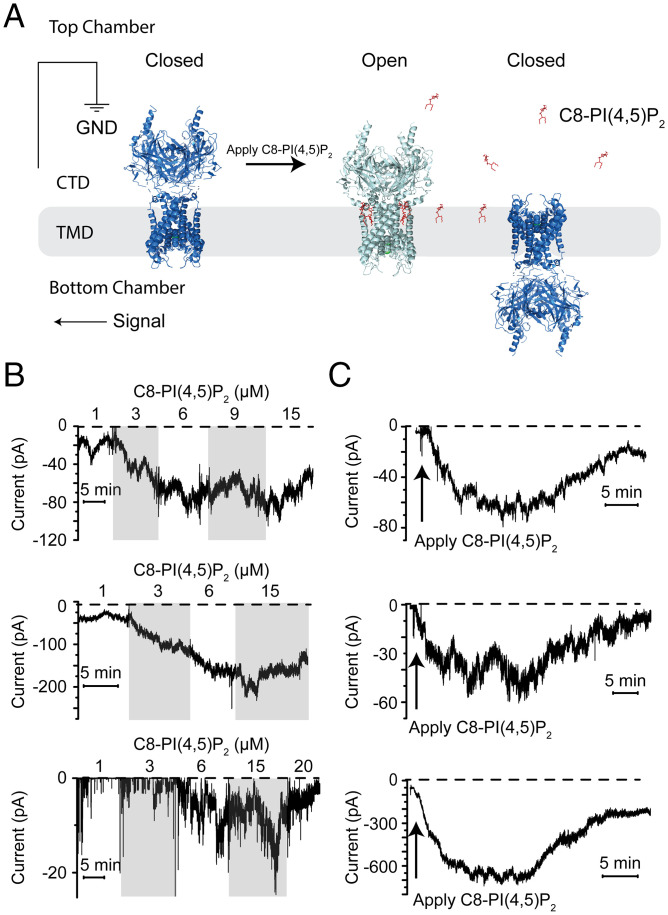
PI(4,5)P_2_-dependent activation of Kir2.2 reconstituted into planar lipid bilayers. (*A*) Schematic of the planar lipid bilayer system. (*B*) Activation of Kir2.2 in response to increasing concentrations of C8-PI(4,5)P_2_ (concentration indicated above) applied to the top chamber. The membrane was held at −100 mV. The current was inverted to follow electrophysiological convention. (*C*) C8-PI(4,5)P_2_–induced current began to decrease spontaneously in about 30 min. Three representative current traces before and after addition of 4 μM C8-PI(4,5)P_2_ to the top chamber are shown. Zero-current level is marked with a dashed line. GND, ground.

X-ray crystallographic and cryoelectron microscopic studies have shown that PI(4,5)P_2_ can modify the conformation of Kir channels (8, 9, 16, 17). For example, in Kir2.2 in the absence of PI(4,5)P_2_, the CTD disengages from the TMD to form a “CTD-undocked” conformation, which is accompanied by a tightly constricted inner helix gate (8). Upon PI(4,5)P_2_ binding, the CTD engages the TMD to form a “CTD-docked” conformation, and the inner helix gate widens (Fig. 1*A*) (16). A similar PI(4,5)P_2_-mediated conformational change was observed in GIRK, and structures determined under varying PI(4,5)P_2_ concentrations indicate that PI(4,5)P_2_ concentrations regulate the equilibrium distribution among CTD-docked and CTD-undocked conformations (17).

Previous electrophysiological studies using inside-out patches from cell membranes showed that two different Kir channels, the KATP channel and a G protein–independent mutant of the GIRK channel, both gated in bursts; that is, intervals of rapid channel opening and closing were separated by long quiescent periods (18, 19). Furthermore, in the GIRK channel, PI(4,5)P_2_ influenced the duration of the burst periods (19). In the KATP channel, PI(4,5)P_2_ influenced the duration of quiescent periods without changing the kinetics within the burst (18). Two other Kir channels, Kir2.1 and Kir2.2, were studied following purification and reconstitution in lipid vesicles (20). Using Rb^+^ flux, Kir2.1 was found to be activated by PI(4,5)P_2_ and PI(3,4,5)P_3_ but not by other phosphatidylinositol lipids.

In this paper, we present an analysis of the Kir2.2 channel gating in response to phosphatidylinositol lipids using the planar lipid bilayer recording system. This system offers complete chemical control of lipid, solution, and protein composition as well as free access to the solution bathing the surfaces of the membrane (21–23). Given that we already have a detailed description of the structural changes that Kir2.2 undergoes upon binding of PI(4,5)P_2_ (8, 16), our goal here was to correlate PI(4,5)P_2_-dependent gating properties with the known structural changes. Furthermore, given our detailed chemical knowledge of the PI(4,5)P_2_ binding site on Kir2.2, we characterized and interpreted the influence of different phosphatidylinositol lipid derivatives on channel gating. We found that competition for the PI(4,5)P_2_-binding site by PI(4)P inhibited Kir2.2 activity. Because both PI(4)P and PI(4,5)P_2_ contribute substantially to the pool of plasma membrane phosphatidylinositol lipids, their competition and interconversion might be relevant to Kir2.2 in vivo (24–26).

## Results

### Dependence of Kir2.2 Opening on C8-PI(4,5)P_2_ Concentration.

Fig. 1*A* shows a schematic of the planar lipid bilayer system used in this study (21–23). A lipid bilayer with a defined composition separates the top and bottom chambers, each filled with electrolyte solution. Liposomes containing Kir2.2 channels are fused with the bilayer, resulting in some channels with the CTD facing the top chamber and others with the CTD facing the bottom chamber (Fig. 1*A*). Soluble reagents such as C8-PI(4,5)P_2_ (PI(4,5)P_2_ with 8-carbon acyl chains) are added to the top chamber. Because C8-PI(4,5)P_2_ is membrane impermeant, only channels with their CTD facing the top chamber are activated (Fig. 1*A*) (23).

We first looked at the dependence of channel activity on PI(4,5)P_2_ concentration. In the absence of C8-PI(4,5)P_2,_ no channel openings were observed. Upon addition of C8-PI(4,5)P_2_, current was increased (Fig. 1 *B* and C), indicating that PI(4,5)P_2_ is both necessary and sufficient for channel opening, as was found previously in a different reconstitution system (20). Kir2.2 activity increased over the C8-PI(4,5)P_2_ concentration range 0 to 15 μM and approached a maximum by 15 μM, as shown in three separate experiments (Fig. 1 *B* and *C*). The analysis of channel activity dependence on C8-PI(4,5)P_2_ was limited by the following property: C8-PI(4,5)P_2_–induced current begins to decrease spontaneously after about 30 min, as shown (Fig. 1*C*). The disappearance of channel activity over time introduced uncertainty to the concentration-dependence of Kir2.2 activity. The disappearance also imposed a limitation on the kinetic analysis; however, as we will demonstrate, this limitation did not prevent us from extracting rate constants using the multichannel analysis described below.

### Influence of C8-PI(4,5)P_2_ on the Gating Kinetics of Kir2.2.

**Fig. 2.**
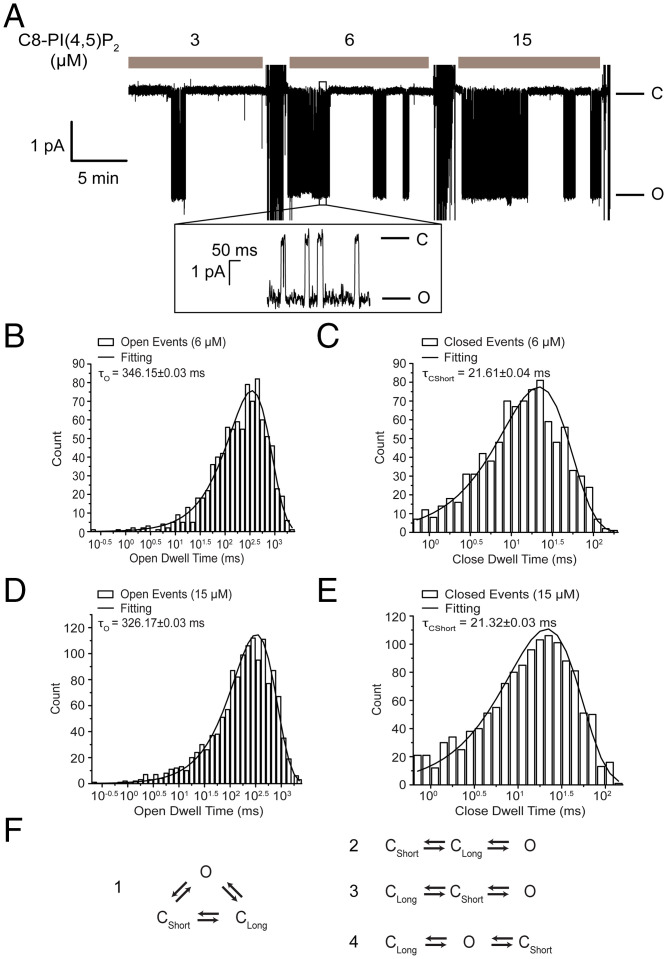
Single-channel analysis of Kir2.2. (*A*) Single-channel recording of Kir2.2 at −100 mV in the presence of 3, 6, and 15 μM C8-PI(4,5)P_2_. An expanded trace is shown in the inset. The open (O) and closed (C) levels are indicated. Large current deflections between adjacent C8-PI(4,5)P_2_ concentrations are due to mixing of the solution after each addition of C8-PI(4,5)P_2_. (*B*) The open dwell-time distribution of Kir2.2 at 6 μM C8-PI(4,5)P_2_ from panel *A* is plotted and fit with a single exponential function (solid line). (*C*) The dwell-time distribution of closed events within the burst at 6 μM C8-PI(4,5)P_2_ from panel *A* is plotted and fit with a single exponential function (solid line). (*D*) The open dwell-time distribution of Kir2.2 at 15 μM C8-PI(4,5)P_2_ from panel *A* is plotted and fit with a single exponential function (solid line). (*E*) The dwell-time distribution of closed events within the burst at 15 μM C8-PI(4,5)P_2_ from panel *A* is plotted and fit with a single exponential function (solid line). (*F*) Connectivity diagram (*1*) and possible linear kinetic subschemes (*2* to *4*) with one short closed state (C_Short_), one C_Long_, and one open state (O).

We next looked at how C8-PI(4,5)P_2_ influences the kinetics of Kir2.2 gating. Fig. 2*A* shows a single-channel trace recorded in the presence of 3, 6, and 15 μM C8-PI(4,5)P_2_. The channel opened in bursts of activity separated by quiescent intervals. We saw from this trace two processes that operated on very different timescales. The relatively fast process, occurring on the subsecond timescale, accounted for rapid channel opening and closing within a burst of activity (Fig. 2*A*, *Inset*). The slow process, occurring on the minute timescale, accounted for the appearance and disappearance of bursts. Lifetime histograms for events within bursts showed single open-time and single closed-time distributions, which corresponded to the relatively rapid gating transitions that occurred within a burst (Fig. 2 *A*–*E*). Note that the histograms were essentially unchanged when the concentration of C8-PI(4,5)P_2_ was increased from 6 μM to 15 μM (compare Fig. 2*B* with Fig. 2*D* and Fig. 2*C* with Fig. 2*E*). This observation indicates that gating transitions within a burst were insensitive to the C8-PI(4,5)P_2_ concentration over a range that influenced open probability (Fig. 1*B*). A second, longer closed dwell time exists because we saw it in the raw trace as long-duration time intervals free of channel activity (Fig. 2*A*); however, the occurrence of this slow process was too low to accumulate enough events during the recording, which was limited in duration owing to the phenomenon of channel disappearance over time. Fig. 2*F* shows the connectivity diagram for two closed and one open state (Fig. 2*F*, *1*). One linear kinetic subscheme, *2*, was incompatible with the channel record because in this case openings would not be interrupted by brief closures. The remaining two linear kinetic subschemes, *3* and *4*, were compatible with the channel record, which cannot distinguish among them.

**Fig. 3.**
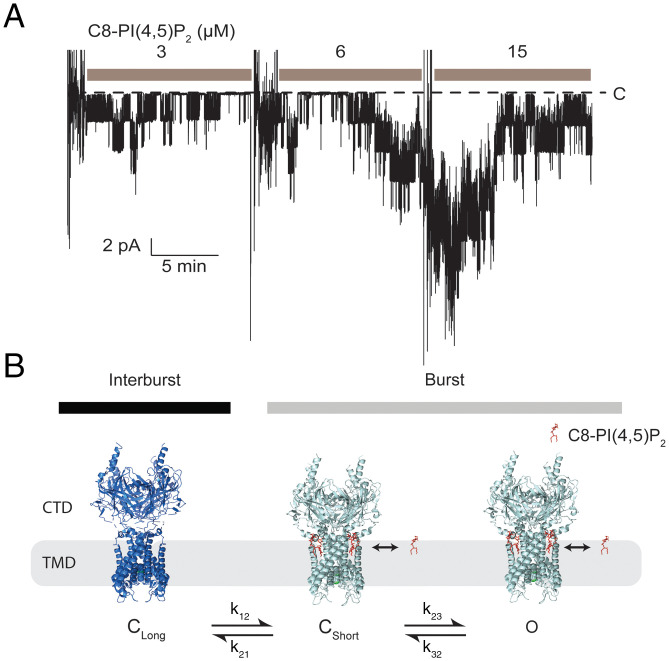
Multi-channel analysis of Kir2.2 at various C8-PI(4,5)P_2_ concentrations. (*A*) Recording from a bilayer with multiple Kir2.2 channels in the presence of 3, 6, and 15 μM C8-PI(4,5)P_2_. Large current deflections between adjacent C8-PI(4,5)P_2_ concentrations are due to mixing of the solution after each addition of C8-PI(4,5)P_2_. The membrane was held at −100 mV. Baseline current level is marked with a dashed line. (*B*) Schematic of the Kir2.2 gating model. The quiescent intervals correspond to the CTD-undocked, closed conformation. After binding a sufficient number of C8-PI(4,5)P_2_, Kir2.2 will enter a burst, which corresponds to the CTD-docked conformation. The slow kinetic process of transition between the burst and quiescent states (k_12_ and k_21_) is sensitive to C8-PI(4,5)P_2_ concentration. Within the burst, the channel can rapidly transit between closed and open states. These rapid gating transitions are insensitive to C8-PI(4,5)P_2_ concentration. After a sufficient number of PI(4,5)P_2_ molecules have bound to enter a burst, at least one more can still bind. Therefore, lipids that bind to the channel can exchange within the burst. C, closed; C_Short_, short closed state; C_Long_, long closed state; O, open state.

In the context of the compatible kinetic schemes, we next asked which transitions are affected by the concentration of C8-PI(4,5)P_2_? Given the low frequency of interburst intervals, confounded by channel disappearance over time, we studied bilayer membranes with several channels present at once. While this approach does not possess the intuitive simplicity of single-channel analysis, it is perfectly valid and, in this case, was enabling because it permitted a sufficient number of events to estimate the rate constants before the channels disappeared. Fig. 3*A* shows a multichannel membrane in the presence of 3, 6, and 15 μM C8-PI(4,5)P_2_. The 15-μM record was used to estimate the total number of channels in the membrane (see *Materials and Methods*), while 3- and 6-μM records were subject to multichannel kinetic analysis (Table 1) (27). The analysis assumes that all channels are identical in their behavior. For most channel types we have studied, including Kir2.2, this assumption is only approximately true. There appeared to be a fraction of outlier channels with lower or higher than average open probability, which undoubtedly contributed to the variation in rate constant values between different experiments reported in Tables 1 and 2. This limitation notwithstanding, Table 1 shows that C8-PI(4,5)P_2_ affected only the rate constants for transitions into and out of long closed state (C_Long_). In detail, when C8-PI(4,5)P_2_ was increased, the burst periods lengthened and the quiescent periods shortened. Consistent with the single-channel trace and histograms in Fig. 2, rate constants for opening and closing within a burst were insensitive to C8-PI(4,5)P_2_ concentration: Within a burst, only the mean number of transitions was affected by C8-PI(4,5)P_2_. Table 1 reports rate constant values for subscheme 3, but subscheme 4 would yield a similar conclusion, that only rate constants into and out of C_Long_ are sensitive to C8-PI(4,5)P_2_ concentration (Fig. 2*F*).

**Table 1.** Kinetics of Kir2.2 gating in the presence of 3 and 6 μM C8-PI(4,5)P_2_

Recording no.	Channel no.	C8-PI(5)P_2_ (μM)	k_12_ (s^−1^)	k_21_ (s^−1^)	k_23_ (s^−1^)	k_32_ (s^−1^)	P(o)	T_burst_ (s)	T_interburst_ (s)	Mean duration of C_S__hort_ (ms)	No. C_S__hort_ per burst
1	8	3	0.0097 ± 0.0007	1.7 ± 0.2	81 ± 2	5.52 ± 0.11	0.097	9.722	105.195	12.05	49
		6	0.021 ± 0.003	1.2 ± 0.2	77 ± 2	5.22 ± 0.08	0.160	13.570	49.146	12.87	65
2	9	3	0.0056 ± 0.0006	2.7 ± 0.4	92 ± 3	6.1 ± 0.2	0.049	6.110	184.571	10.51	34
		6	0.017 ± 0.004	0.8 ± 0.2	84 ± 2	4.85 ± 0.07	0.266	22.669	59.364	11.83	103
3	4	3	0.0054 ± 0.0014	7 ± 2	77 ± 7	6.3 ± 0.5	0.015	2.140	201.661	11.95	12
		6	0.020 ± 0.003	1.2 ± 0.2	85 ± 4	5.3 ± 0.2	0.112	15.136	50.156	11.58	74

Kinetics of Kir2.2 gating based on subscheme 3 (C_Long_ == C_S__hort_ == O) in the presence of 3 and 6 μM C8-PI(4,5)P_2_. Rate constant (k), open probability [P(o)], mean burst duration (T_burst_), mean interburst interval (T_interburst_), mean duration of C_Short_ state, and mean number of short closures per burst (No. C_Short_ per burst) were calculated from three different recordings.

**Table 2.** Kinetics of the C8-PI(4)P competition

Recording no.	Channel no.	C8-PI(4,5)P_2_ (μM)	C8-PI(4)P (μM)	k_12_ (s^−1^)	k_21_ (s^−1^)	k_23_ (s^−1^)	k_32_ (s^−1^)	P(o)	T_burst_ (s)	T_interburst_ (s)	Mean duration of C_Short_ (ms)	No. C_Short_ per burst
1	5	6	0	0.013 ± 0.002	0.90 ± 0.14	52 ± 2	4.20 ± 0.11	0.134	14.907	77.772	19.04	57
			6	0.005 ± 0.002	13 ± 4	57 ± 8	3.0 ± 0.5	0.006	1.865	266.014	14.36	4
2	4	6	0	0.007 ± 0.002	0.62 ± 0.13	53 ± 2	3.35 ± 0.09	0.170	27.597	153.769	18.59	86
			6	0.0013 ± 0.0003	1.8 ± 0.4	43 ± 2	3.7 ± 0.2	0.046	7.475	784.335	22.26	25
3	5	6	0	0.0027 ± 0.0007	0.14 ± 0.05	43.9 ± 1.2	3.72 ± 0.08	0.235	94.889	370.114	22.72	324
			6	0.0005 ± 0.0005	0.39 ± 0.09	42.1 ± 1.2	3.03 ± 0.08	0.117	38.427	2,271.968	23.54	108

Kinetics of Kir2.2 gating based on subscheme 3 (C_Long_ == C_Short_ == O) in the presence of 6 μM C8-PI(4,5)P_2_ before and after application of 6 μM C8-PI(4)P. Rate constant (k), open probability [P(o)], mean burst duration (T_burst_), mean interburst interval (T_interburst_), mean duration of C_Short_ state, and mean number of short closures per burst (No. C_Short_ per burst) were calculated from three different recordings.

The functional analysis leads to the simple conclusion that only the slow kinetic process of transition between the burst and quiescent states is sensitive to C8-PI(4,5)P_2_. The structural studies showed that the binding of C8-PI(4,5)P_2_ was associated with a large conformational change between the CTD-undocked and CTD-docked structures (8, 16, 17). We thus proposed that the slow gating transitions in the channel recordings correspond to CTD engagement and disengagement and that when the CTD is engaged, the pore can open (Fig. 3*B*). The rapid gating transitions within a burst would then represent C8-PI(4,5)P_2_–independent conformational changes that occur elsewhere along the ion conduction pathway. According to this model, the channel record provides a dynamic readout of the CTD engagement and disengagement process, whose equilibrium is shifted by the C8-PI(4,5)P_2_ concentration.

### Effect of Phosphatidylinositol Lipid Derivatives on Gating.

**Fig. 4.**
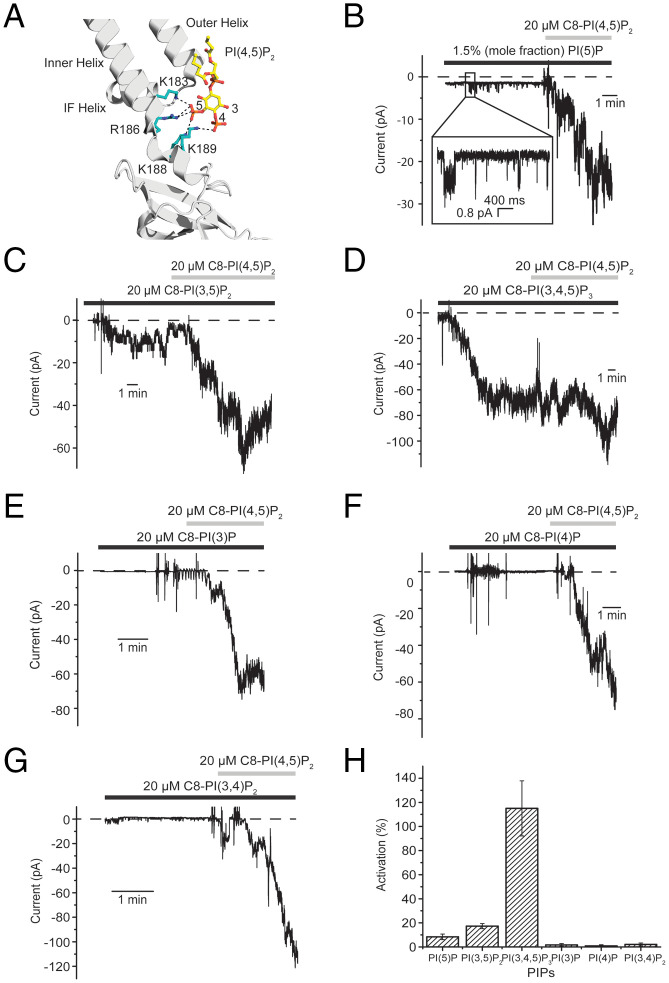
Effect of different phosphatidylinositol lipids on Kir2.2. (*A*) C8-PI(4,5)P_2_–binding site on Kir2.2 (PDB 3SPH). The channel is shown as a gray ribbon. C8-PI(4,5)P_2_ is shown as sticks and is colored according to atom type: oxygen, red; phosphorus, orange; and carbon, yellow. Sidechains of residues that form hydrogen bonds with 4′ or 5′ phosphate are shown as sticks and are colored teal. IF helix, interfacial helix. Data for Fig. 4*A* adapted from Hansen *et al*. (16). (*B*) PI(5)P can activate Kir2.2. Short openings (inset) were observed from membranes with 1.5% (mole fraction) PI(5)P. Further application of 20 μM C8-PI(4,5)P_2_ resulted in a current increase. The membrane was held at −100 mV. (*C*) C8-PI(3,5)P_2_ can activate Kir2.2. Further application of 20 μM C8-PI(4,5)P_2_ resulted in a significant increase of current. The membrane was held at −100 mV. (*D*) C8-PI(3,4,5)P_3_ activates Kir2.2 to a similar extent as C8-PI(4,5)P_2_. The membrane was held at −100 mV. (*E*–*G*) 20 μM C8-PI(3)P (*E*), C8-PI(4)P (*F*), or C8-PI(3,4)P_2_ (*G*) failed to activate Kir2.2 despite the presence of Kir2.2 channels in the membrane, demonstrated by further addition of 20 μM C8-PI(4,5)P_2_. The membranes were held at −100 mV. Large current deflections were caused by mixing. Zero-current level is marked with a dashed line. (*H*) Summary of the effect of various phosphatidylinositol lipids on Kir2.2 gating. Here, the activation was calculated as the ratio of average current induced by 20 μM of the indicated short-chain phosphatidylinositol lipid (or 1.5% long-chain PI(5)P) to the average current following subsequent addition of 20 μM C8-PI(4,5)P_2_ (i.e., I_indicated PIP_/I_C8-PI(4,5)P2 + indicated PIP_, mean ± SEM, *n* = 3). PIP, phosphatidylinositol lipid.

Earlier studies showed that phosphatidylinositol lipids with different phosphate substitutions can also interact with Kir channels, including Kir2.2 (26). Fig. 4*A* shows the main, direct chemical interactions between C8-PI(4,5)P_2_ and Kir2.2 derived from the crystal structure (16). Individual membrane recordings using the planar bilayer system show the effects of several different phosphatidylinositol lipids (Fig. 4 *B*–*G*). During each recording, application of the lipid under examination was followed by application of C8-PI(4,5)P_2_ to ensure the presence of Kir2.2 channels in the membrane. Soluble (C8) phosphatidylinositol lipids were used except for PI(5)P, which was only available in a long acyl-chain form and thus was included as part of the membrane’s lipid composition. We found that only phosphatidylinositol lipids with the 5′ phosphate could activate the Kir2.2 channel, and the magnitude of Kir2.2 activation for the lipid concentrations applied was PI(4,5)P_2_ ∼ PI(3,4,5)P_3_ > PI(3,5)P_2_ > PI(5)P (Fig. 4 *B*–*D* and *H*). Essentially no activation was observed with PI(3)P, PI(4)P, and PI(3,4)P_2_ (Fig. 4 *E*–*G* and *H*). The results are consistent with a previous study showing that Kir2.2 can be activated in membrane patches from cells by C8-PI(4,5)P_2_ and C8-PI(3,4,5)P_3_, but not C8-PI(3,4)P_2_ (28). In the crystal structure of Kir2.2, the 5′ phosphate forms ionized hydrogen bonds with several basic residues at and near the base of the inner helix, which forms the gate (Fig. 4*A*) (16). This critical location of the 5′ phosphate in the crystal structure is compatible with its functional requirement to open the Kir2.2 channel.

**Fig. 5.**
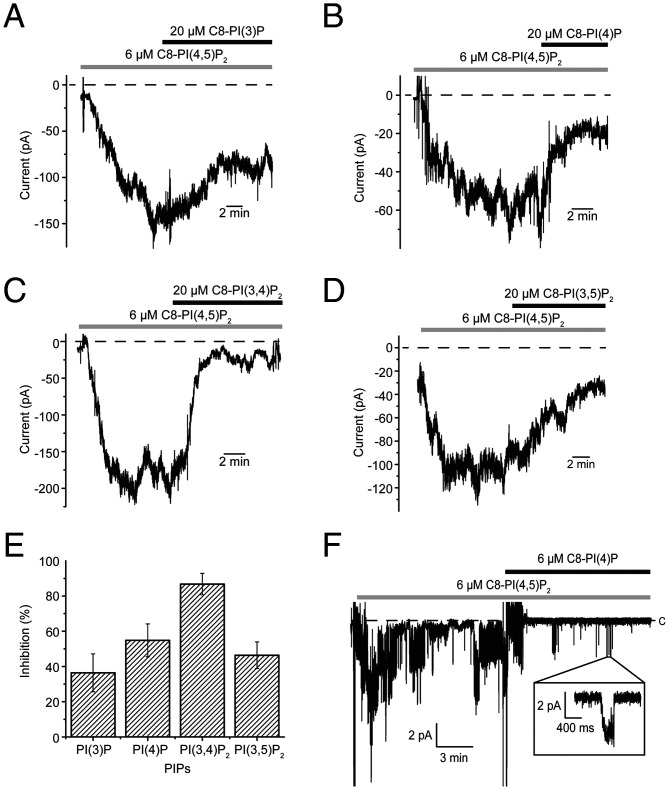
Competitive inhibition of C8-PI(4,5)P_2_ activation by various phosphatidylinositol lipids. (*A*–*D*) C8-PI(3)P (*A*), C8-PI(4)P (*B*), C8-PI(3,4)P_2_ (*C*), or C8-PI(3,5)P_2_ (*D*) inhibited Kir2.2 currents that were activated by C8-PI(4,5)P_2_. Following Kir2.2 activation by 6 μM C8-PI(4,5)P_2_, application of 20 μM C8-PI(3)P (*A*), C8-PI(4)P (*B*), C8-PI(3,4)P_2_ (*C*), or C8-PI(3,5)P_2_ (*D*) caused a reduction in current. The membranes were held at −100 mV. Zero-current level is marked with a dashed line. (*E*) Summary of the inhibition by different phosphatidylinositol lipids. Percentages of current decrease after application of 20 μM of the indicated phosphatidylinositol lipids are plotted (i.e., I_decreased current_/I_6 μM C8-PI(4,5)P2_, mean ± SEM, *n* = 3). (*F*) Current recorded from a bilayer containing multiple Kir2.2 channels after application of 6 μM C8-PI(4,5)P_2_ followed by additional 6 μM C8-PI(4)P. An expanded trace is shown in the inset. The membrane was held at −100 mV. C, closed; PIP, phosphatidylinositol lipid.

Do phosphatidylinositol lipids that do not activate Kir2.2 fail to bind altogether, or do they bind but fail to open the gate? An earlier work based on a flux assay with Kir2.1 (29) and the data in Fig. 5 for Kir2.2 suggest the latter, that certain phosphatidylinositol lipids inhibit Kir2.2 activation by competing with PI(4,5)P_2_ for its binding site. Following Kir2.2 activation by 6 μM C8-PI(4,5)P_2_, addition of 20 μM C8-PI(3)P, PI(4)P, or PI(3,4)P_2_ caused a pronounced reduction in current (Fig. 5 *A*–C and *E*). PI(3,5)P_2_, which itself activates Kir2.2 but to a lesser extent than PI(4,5)P_2_, also reduced current, apparently by competing for the site (Fig. 5 *D* and *E*). Because of this competition, we note that the channel responses to PI(4,5)P_2_ in Figure 4 were blunted in the presence of competing lipids. In membranes with a small number of channels, we tried to determine how a competing lipid, C8-PI(4)P, influences the kinetics of gating (Fig. 5*F* and Table 2). Again, only the rate constants into and out of C_Long_ were affected, as if addition of C8-PI(4)P mimics a reduction in the concentration of C8-PI(4,5)P_2_.

## Discussion

These results build on previous studies showing that PI(4,5)P_2_ is necessary and sufficient to open Kir2.2 (20). They also build on earlier characterizations of phosphatidylinositol lipid specificity in the activation of Kir channels in general (26). The present study advances our understanding by analyzing the phosphatidylinositol lipid-dependent gating of Kir2.2 in the compositionally defined lipid bilayer system. It also presents a mechanistic model derived by correlating C8-PI(4,5)P_2_–dependent changes in the atomic structure of Kir2.2 with C8-PI(4,5)P_2_–dependent changes in the kinetics of gating. The accuracy of the kinetic data is limited for reasons described above. Nevertheless, the conclusion that C8-PI(4,5)P_2_ influences only the relatively slow transitions that govern exchange between the burst and quiescent periods is robust. Furthermore, C8-PI(4,5)P_2_ influenced both the rates of entry and exit from the long quiescent periods for Kir2 channels. Given that Kir2.2 is the most thoroughly understood Kir2 channel from a structural point of view, this kinetic analysis has value. In the model, we connected the large conformational change observed in structural studies—C8-PI(4,5)P_2_–mediated engagement between the CTD and TMD—with the very slow burst-quiescent period interconversions. The magnitude of conformational change documented in the structure was compatible with slow kinetic transitions. This idea is depicted in cartoon form in Fig. 3*B*. Of course, this is a hypothesis and not a claim that the model is necessarily true. In the CTD-docked conformation, the pore opens and closes rapidly in a C8-PI(4,5)P_2_–independent manner. Other Kir channels also exhibit burst kinetics (18, 19), and in KATP, ATP-independent gating within burst periods has been attributed to processes inside the selectivity filter (30). The basis for rapid gating in Kir2.2 is still unknown.

A G protein–independent GIRK channel was found to exhibit a more complex dependence on PI(4,5)P_2_ than we describe here for Kir2.2 (19). In the mutant GIRK channel, in addition to influencing the burst (but not the interburst) duration, multiple open states were deduced and attributed to different degrees of PI(4,5)P_2_ occupation. In Kir2.2, we observed only a single open state and can say only the following regarding the functional stoichiometry of PI(4,5)P_2_ activation: 1) that the quiescent periods shortened with increasing PI(4,5)P_2_ concentration, together with no current observed in the absence of PI(4,5)P_2_, suggests that at least one bound PI(4,5)P_2_ molecule is required to enter a burst and 2) that the burst periods lengthened with increasing PI(4,5)P_2_ concentration suggests that after a sufficient number of PI(4,5)P_2_ molecules have bound to enter a burst, at least one more PI(4,5)P_2_ can still bind (Fig. 3*B*). If this were not the case, then the burst duration would be independent of the PI(4,5)P_2_ concentration. In conclusion, somewhere between one and three PI(4,5)P_2_ molecules would seem to be required to stabilize the structure underlying the burst state, which we propose is the CTD-docked state. We know from the structures that four PI(4,5)P_2_ molecules can bind to the CTD-docked conformation, but if the model is correct, fewer than four can support the CTD-docked conformation.

The requirement of a 5′ phosphate to achieve channel opening seems compatible with the crystal structure of Kir2.2 with PI(4,5)P_2_ bound because the 5′ phosphate interacts directly with amino acids on the inner helix, which forms the gate. PI(4,5)P_2_ also makes other interactions with the channel, so it is understandable that a phosphatidylinositol lipid without the 5′ phosphate would still bind to the site. This is undoubtedly why PI(4)P, for example, competes with PI(4,5)P_2_ for the phosphatidylinositol lipid–binding site on the channel. PI(4)P as a competing lipid is particularly interesting because, along with PI(4,5)P_2_, it is abundant in the plasma membrane (25). Moreover, PI(4)P is a precursor in the synthesis of PI(4,5)P_2_, and dephosphorylation of PI(4,5)P_2_ generates PI(4)P (24–26). Because PI(4,5)P_2_ activates and PI(4)P competitively inhibits, changes in lipid metabolism could give rise to a very steep change in the level of Kir2.2 activity.

## Materials and Methods

### Cloning, Expression, and Purification.

A synthetic gene fragment (Bio Basic, Inc.) encoding residues 38 to 369 of chicken Kir2.2 (cKir2.2) channel (GI:118097849) was subcloned into a modified pEG BacMam vector with a C-terminal green fluorescent protein (GFP)–1D4 tag linked by a preScission protease site (31). This construct was used in all experiments in this study.

Bacmid containing cKir2.2 gene was generated according to the manufacturer’s instructions (Invitrogen) by transforming the cKir2.2 pEG BacMam construct into *Escherichia coli* DH10Bac cells. The bacmid was then transfected into *Spodoptera frugiperda* Sf9 cells to produce baculoviruses using Cellfectin II (Invitrogen). After two rounds of amplification, P3 viruses (1:10 v:v ratio) were added to suspension cultures of HEK293S GnTI^−^ cells (American Type Culture Collection) at a density around 1.5 to 3 × 10^6^ cells/mL in Freestyle 293 media (GIBCO) supplemented with 2% fetal bovine serum (GIBCO) at 37 °C for protein expression. After the infected cells were inoculated for 20 h at 37 °C, 10 mM sodium butyrate was added, and the temperature was changed to 30 °C. Cells were harvested ∼40 h after the temperature was changed (31).

A 4-L cell pellet was first resuspended in 200 mL hypotonic lysis buffer (20 mM Tris⋅HCl, pH 8, 1 mM EDTA, 1 mM phenylmethylsulfonyl fluoride [PMSF], 0.1 mg/mL 4-(2-Aminoethyl) benzenesulfonyl fluoride hydrochloride [AEBSF], 0.1 mg/mL soybean trypsin inhibitor, 1 mM benzamidine, 1 μg/mL pepstatin A, 1 μg/mL leupeptin, 5 μg/mL aprotinin, and 0.02 mg/mL deoxyribonuclease [Dnase] I) at 4 **°**C. The lysate was then centrifuged, and the pellet was homogenized using a Dounce homogenizer with 100 mL extraction buffer containing 20 mM Tris⋅HCl, pH 8, 1 mM EDTA, 320 mM KCl, 2 mM PMSF, 0.1 mg/mL AEBSF, 0.1 mg/mL soybean trypsin inhibitor, 1 mM benzamidine, 1 μg/mL pepstatin A, 1 μg/mL leupeptin, 5 μg/mL aprotinin, and 0.1 mg/mL Dnase I. The lysate was supplemented with 40 mM n-Decyl-β-D-Maltopyranoside (DM) to extract at 4 **°**C for 1 to 2 h and then centrifuged. The supernatant was incubated with GFP nanobody-conjugated affinity resin (CNBr-activated Sepharose 4B resin from GE Healthcare) by rotating at 4 **°**C for 1 h. Resin was then washed with 10 column volumes of wash buffer (20 mM Tris⋅HCl, pH 8, 1 mM EDTA, 150 mM KCl, and 6 mM DM). PreScission protease (∼1:20 w:w ratio) was then added for digestion overnight by rotating. Flow through was collected, concentrated, and loaded onto Superdex 200 increase size exclusion column (GE Healthcare) equilibrated in 20 mM Tris⋅HCl, pH 8, 1 mM EDTA, 150 mM KCl, 4 mM DM, 10 mM dithiothreitol (DTT), and 2 mM Tris(2-carboxyethyl)phosphine hydrochloride [TCEP]. The purified protein was then concentrated for reconstitution.

### Reconstitution of Proteoliposomes.

The reconstitution was performed as previously described (23, 32–35) with minor modifications. Briefly, a lipid mixture composed of 3:1 (w:w) 1-palmitoyl-2-oleoyl-sn-glycero-3-phosphoethanolamine:1-palmitoyl-2-oleoyl-sn-glycero-3-phospho-(1′-rac-glycerol) (Avanti) was dried under Argon, rehydrated in reconstitution buffer (10 mM potassium phosphate, pH 7.4, 150 mM KCl, 1 mM EDTA, and 3 mM DTT) to 20 mg/mL by rotating for 20 min at room temperature followed by sonication with a bath sonicator. 1% DM was then added. The lipid mixture was rotated for 30 min and sonicated again till clear. Equal volumes of protein (at 2 mg/mL and 0.2 mg/mL) and lipid (at 20 mg/mL) were mixed, resulting in protein:lipid (w:w) ratios of 1:10 and 1:100, respectively. The mixture was incubated at 4 °C for 1 h and then dialyzed against 2 L reconstitution buffer for 2 d, exchanging buffer every 12 h. Biobeads were added to the reconstitution buffer for the last 12 h. The resulting proteoliposomes were frozen with liquid nitrogen and stored at −80 °C.

### Electrophysiology.

The bilayer experiments were performed as previously described with minor modifications (23, 36). A piece of polyethylene terephthalate transparency film separated the two chambers of a polyoxymethylene block, which were filled with buffer containing 10 mM potassium phosphate, pH 7.4, 150 mM KCl, and 2 mM MgCl_2_.

Decane-lipid mixture of 1,2-dioleoyl- sn-glycero-3-phosphoethanolamine:1-palmitoyl-2-oleoyl-sn-glycero-3-phosphocholine:1-palmitoyl-2-oleoyl-sn-glycero-3-phospho-L-serine (Avanti) (w:w:w 2:1:1) at 20 mg/mL was prepainted over an ∼100-μm hole on the transparency film. Voltage was controlled with an Axopatch 200B amplifier in whole-cell mode. The analog current signal was low-pass filtered at 1 kHz (Bessel) and digitized at 10 kHz with a Digidata 1322A digitizer. Digitized data were recorded on a computer using the software pClamp (Molecular Devices, Sunnyvale, CA). Experiments were performed at room temperature. For macroscopic current recordings, data reduction with a reduction factor of 100 and 5-Hz Gaussian low-pass filter was applied for plotting purposes. For single-channel recordings, a 300-Hz Gaussian low-pass filter was applied to the expanded trace (Fig. 2*A*, *Inset*), or data reduction with a reduction factor of 200 was applied to the whole single-channel recording for plotting.

### Kinetic Analysis.

Recordings containing one to nine channels were idealized through half-amplitude threshold crossing and analyzed using Clampfit software (Molecular Devices).

For single-channel recordings, open or closed dwell-time distributions for events within the burst were fitted to an exponential probability density function. Models with different term numbers were compared, and the best model was selected (37).

**Scheme 1.**
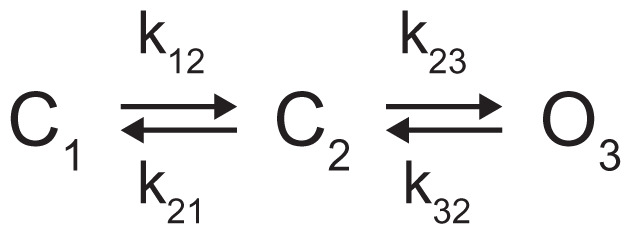
Three-state linear scheme. The closed states are denoted as C_1_ and C_2_, and the open state is denoted as O_3_. Rate constants between these states are denoted as k_12_, k_21_, k_23_, and k_32_.

Multichannel recordings were analyzed as described (27). Event lists were fitted using a three-state linear scheme (Scheme 1). Dead time was 0.27 ms. Channel numbers used in the multichannel kinetic analysis were estimated using the maximum number of observed open channel levels (from 15 μM C8-PI(4,5)P_2_ recordings). Rate constants between all states (k_12_, k_21_, k_23_, and k_32_) were obtained by simultaneous fit to the dwell-time histograms of all conductance levels (27). The mean number of short closures per burst, mean burst, and interburst duration were then calculated based on the rate constants − mean number of short closures per burst $=\frac{{\text{k}}_{23}}{{\text{k}}_{21}}$ , mean interburst duration $=\frac{{\text{k}}_{23}+{\text{k}}_{21}+{\text{k}}_{12}}{{\text{k}}_{12}{*\text{k}}_{23}}+\frac{1}{{\text{k}}_{21}+{\text{k}}_{23}}\;$ , and mean burst duration $=\frac{1}{{\text{k}}_{21}}*\left(\frac{{\text{k}}_{23}+{\text{k}}_{21}}{{\text{k}}_{32}}+\frac{{\text{k}}_{23}}{{\text{k}}_{21}+{\text{k}}_{23}}\right)$ . Under our condition, that is ${\text{k}}_{23}\gg ({\text{k}}_{21}+{\text{k}}_{12})$ , mean interburst duration can be approximated as $\frac{1}{{\text{k}}_{12}}$ , and mean burst duration can be approximated as $\frac{1}{{\text{k}}_{21}}*\left(1+\frac{{\text{k}}_{23}}{{\text{k}}_{32}}\right)$ .

### Estimation of the Channel Number Used in the Multichannel Kinetic Analysis.

**Scheme 2.**
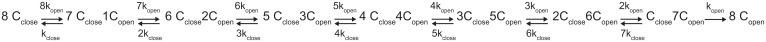
This scheme, with the rate constants weighted as shown, models a membrane with *n* = 8 identical channels. The quiescent state of each channel is denoted as C_close_, and the burst is denoted as C_open_. The leftmost state represents the membrane with eight closed channels. The rightmost state represents the membrane with eight open (i.e., burst) channels.

To evaluate the validity of using the maximum number of observed open channel levels (from 15 μM C8-PI(4,5)P_2_ recordings) as the channel number (N) in the multichannel kinetic analysis, we asked what is the probability of observing N channels at least once during the duration of the record? This probability is a function of N, the rate constants, and the initial condition. This probability is given by the integral of the first passage time probability density function for the appearance of the rightmost state for the scheme, for *n* = 8, shown in Scheme 2.

Using Mathematica (Wolfram), we estimated that the mean first passage time was 268 s and that the probability of observing eight channels at least once during the duration of the record (10 min) was 0.9. Given the 10% chance that we could not see all eight channels open within 10 min and the problem of channel disappearance over time, it is possible that we underestimated the channel number. We therefore asked, if we assign the incorrect value for N, will our conclusion that C8-PI(4,5)P_2_ affects only the burst and interburst periods be wrong? Using recording 3 (Table 1), in which we assigned *n* = 4 on the basis of direct observation, we reanalyzed the record for *n* = 4 to 10 (Table 3). The value of N had little influence on the determination of kinetic values within the burst and had a small influence on k_21_ and mean burst duration. The main effect was on k_12_ and mean interburst duration. However, importantly, when C8-PI(4,5)P_2_ was increased, the burst periods lengthened and the quiescent periods shortened. Thus, our general conclusion that C8-PI(4,5)P_2_ concentrations influenced the transitions between the burst and interburst states holds even if we underestimated N.

**Table 3.** Reanalysis of recording 3 from Table 1, using various channel numbers (N)

Recording no.	Channel no.	C8-PI(4,5)P_2_ (µM)	k_12_ (s^−1^)	k_21_ (s^−1^)	k_23_ (s^−1^)	k_32_ (s^−1^)	P(o)	T_burst_ (s)	T_interburst_ (s)	Mean duration of C_Short_ (ms)	No. C_S__hort_ per burst
3	4	3	0.0054 ± 0.0014	7 ± 2	77 ± 7	6.3 ± 0.5	0.015	2.140	201.661	11.95	11.52
		6	0.020 ± 0.003	1.2 ± 0.2	85 ± 4	5.3 ± 0.2	0.112	15.136	50.156	11.58	73.92
	5	3	0.0044 ± 0.0016	7 ± 2	75.8 ± 7.1	6.2 ± 0.6	0.0117	2.110	246.782	12.12	11.30
		6	0.016 ± 0.003	1.3 ± 0.3	86 ± 4	5.33 ± 0.19	0.0892	13.353	63.729	11.43	66.13
	6	3	0.0036 ± 0.0014	7 ± 2	76 ± 7	6.2 ± 0.6	0.0098	2.096	300.129	12.12	11.22
		6	0.013 ± 0.003	1.4 ± 0.3	87 ± 4	5.4 ± 0.2	0.0743	12.551	78.305	11.37	62.42
	7	3	0.0031 ± 0.0009	7 ± 2	76 ± 7	6.2 ± 0.5	0.0084	2.085	353.833	12.12	11.15
		6	0.011 ± 0.002	1.4 ± 0.3	87 ± 4	5.37 ± 0.17	0.0637	12.036	92.779	11.33	60.01
	8	3	0.0027 ± 0.0013	7 ± 2	76 ± 7	6.2 ± 0.5	0.0073	2.077	407.228	12.12	11.11
		6	0.0095 ± 0.0015	1.5 ± 0.3	87 ± 4	5.39 ± 0.18	0.0557	11.678	107.161	11.31	58.34
	9	3	0.0024 ± 0.0006	7 ± 2	76 ± 7	6.2 ± 0.6	0.0065	2.072	460.095	12.12	11.08
		6	0.0084 ± 0.0016	1.5 ± 0.3	87 ± 4	5.4 ± 0.2	0.0496	11.438	121.653	11.27	57.25
	10	3	0.0021 ± 0.0009	7 ± 2	76 ± 7	6.2 ± 0.6	0.0059	2.067	513.606	12.12	11.05
		6	0.0075 ± 0.0015	1.5 ± 0.3	87 ± 4	5.4 ± 0.2	0.0446	11.247	136.103	11.26	56.36

Kinetics of Kir2.2 gating based on subscheme 3 (C_Long_ == C_Short_ == O) in the presence of 3 and 6 μM C8-PI(4,5)P2. Rate constant (k), open probability [P(o)], mean burst duration (T_burst_), mean interburst interval (T_interburst_), mean duration of C_Short_ state, and mean number of short closures per burst (No. C_Short_ per burst) were calculated from recording 3 from Table 1 using the channel numbers listed in column 2.

## Data Availability

All study data are included in the main text.

## Change History

May 5, 2022: The text of this article has been updated.
